# Estimation of tsunami direction and horizontal velocity field from tsunami magnetic field

**DOI:** 10.1098/rsta.2024.0083

**Published:** 2024-12-02

**Authors:** Zhiheng Lin

**Affiliations:** ^1^ Center for Data Assimilation Research and Applications, Joint Support-Center for Data Science Research, Tachikawa, Japan; ^2^ The Institute of Statistical Mathematics, Tachikawa, Japan

**Keywords:** tsunami early warning, tsunami magnetic field, tsunami velocity field

## Abstract

During tsunamis, the interaction between moving seawater and the Earth’s magnetic field generates a magnetic field detectable by electromagnetic sensors located on land or on the seafloor. In this study, we introduce new methods for estimating tsunami propagation direction and horizontal velocity fields using tsunami magnetic field data. We derive a transfer function that establishes a relationship between the tsunami magnetic field and the velocity field, emphasizing the alignment between the horizontal magnetic field and the tsunami’s propagation direction. This transfer function was validated with data from the 2009 Samoa and 2010 Chile tsunamis. Our findings show that tsunami horizontal velocities and directions can be accurately determined from these magnetic fields. This advancement enables the use of tsunami magnetic fields to provide comprehensive data for tsunami warning systems and to improve the inversion of tsunami seismic sources.

This article is part of the theme issue ‘Magnetometric remote sensing of Earth and planetary oceans’.

## 1. Introduction

A tsunami is generated when seawater is uplifted and spreads outwards owing to earthquakes, underwater volcanic eruptions or underwater landslides. Upon reaching the coastline, tsunamis can cause significant destruction. To prevent and mitigate tsunami hazards, warning systems have been established that use tsunameters on the seafloor and onshore. The most effective tsunami warning systems rely on underwater pressure sensors [1], such as the USA’s DART system [2], Japan’s DONET [3] and S-net [4]. These pressure sensors can detect changes in sea-surface pressure during tsunami propagation. However, seafloor pressure gauges cannot directly measure the direction and speed of a tsunami, which means that the warning systems must wait until multiple stations have recorded the tsunami before making a reliable prediction.

As tsunamis propagate, the movement of seawater interacts with the Earth’s magnetic field, creating an induced magnetic field. This tsunami-induced magnetic field (hereafter called tsunami magnetic field) can be detected using multi-component electromagnetic sensors, either on land or on the seafloor [5–7]. From the tsunami magnetic field, tsunami sea-level changes can be calculated with greater precision than that of underwater pressure sensors [8–11]. Additionally, the horizontal direction of the tsunami magnetic field aligns with the direction of tsunami propagation, allowing for the determination of tsunami motion direction from the magnetic field [6]. However, the accuracy of tsunami direction estimates obtained from tsunami magnetic fields has not yet been thoroughly examined.

Different from underwater pressure sensors, underwater electromagnetic sensors capture three-dimensional tsunami signals. This capability allows for the extraction of multi-dimensional tsunami information, such as tsunami velocity fields, from the magnetic field signals. While tsunami sea-level changes provide valuable information, tsunami velocity fields offer additional insights, including the intensity of the tsunami and variations in the direction of its propagation. Incorporating tsunami velocity data into tsunami warning systems has the potential to enhance both the accuracy and efficiency of tsunami predictions. For this purpose, developing methods to obtain tsunami velocity fields from tsunami magnetic fields is essential.

Therefore, the goal of this paper is to develop a method for determining tsunami direction and velocity fields from tsunami magnetic fields. We first derive the relationship between the tsunami magnetic field, tsunami direction and tsunami velocity field using Maxwell’s equations. By analysing both measured tsunami magnetic field data and simulated tsunami velocity field data, we investigate the connection between tsunami magnetic fields and tsunami motion. Finally, we use Monte Carlo simulations to evaluate the accuracy of estimating tsunami direction and velocity from tsunami magnetic fields.

## 2. Equations

### (a) Equations for estimating tsunami propagation direction

**Figure 1.**
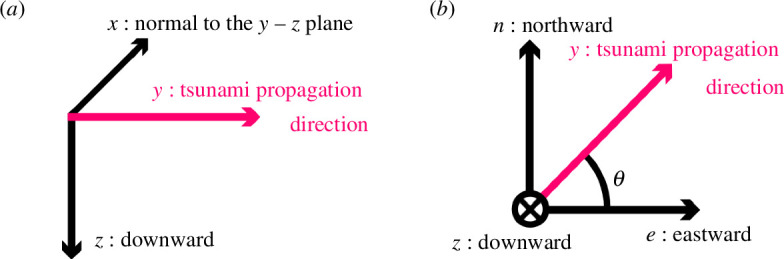
The *x–y–z* (*a*) and *n–e–z* (*b*) coordinates

Before deriving the equations, we clarify the coordinate systems used in this paper. Two right-hand Cartesian coordinate systems are employed. The first is the *x–y–z* coordinate system, where the *y*-direction is the same direction as tsunami propagation, the *x*-direction is perpendicular to the *y*-direction on the horizontal plane and the *z*-direction points downward (figure 1*a*). This coordinate system simplifies the tsunami wave equations. The second is the *n–e–z* coordinate system, it is a geography-related coordinate system in which the *n*- and *e*-directions are the directions to geographical north and east, respectively, and the *z*-direction (in common with the *x–y–z* system) points downward (figure 1*b*). The equations will be rotated into this coordinate system for easier application to actual observational data.

The equation of the motion-induced magnetic field generated by a long wave (which includes tsunamis) in the ocean is [12]


(2.1) $$\begin{array}{r} \begin{array}{r} \nabla^{2}b=\sigma_{oc}\mu_{0}\frac{\partial b}{\partial t}-\sigma_{oc}\mu_{0}\nabla \times (V\times F), \end{array} \end{array}$$

where $\sigma_{oc}$ and $\mu_{0}$ are the conductivity of seawater and the permeability of free space, respectively. This equation indicates that the seawater particles moved by a tsunami (with tsunami velocity V) cut the Earth’s ambient magnetic field, F, generating an induced-magnetic field, b. Here, we ignore the temporal and spatial variations of the Earth’s magnetic field, which means F is a fixed parameter in our calculation. In the *x–y–z* coordinate system equation (2.1) has the form


(2.2) $$\begin{array}{r} \begin{array}{r} \left\{ \begin{array}{ll} & b_{x}=0\\ \left(\frac{\partial^{2}}{\partial y^{2}}+\frac{\partial^{2}}{\partial z^{2}}\right) & b_{y}=\sigma_{oc}\mu_{0}\frac{\partial b_{y}}{\partial t}+\sigma_{oc}\mu_{0}\frac{\partial }{\partial z}(-v_{y}F_{z}+v_{z}F_{y})\\ \left(\frac{\partial^{2}}{\partial y^{2}}+\frac{\partial^{2}}{\partial z^{2}}\right) & b_{z}=\sigma_{oc}\mu_{0}\frac{\partial b_{z}}{\partial t}+\sigma_{oc}\mu_{0}\frac{\partial }{\partial y}(v_{y}F_{z}-v_{z}F_{y}), \end{array}\right. \end{array} \end{array}$$

where the tsunami velocity field is given by $V=(0,v_{y},v_{z})$, representing a simple two-dimensional wave. Consequently, the Laplacian operator simplifies to $\frac{\partial^{2}}{\partial y^{2}}+\frac{\partial^{2}}{\partial z^{2}}$, since $\frac{\partial^{2}}{\partial x^{2}}=0$ owing to the absence of variation in the *x*-direction. Equation (2.2) can be easily solved if the tsunami velocity field is represented by a two-dimensional linear dispersion wave [9,10,13]. However, this equation cannot be used without information on the tsunami’s direction, as it relies on an *x–y–z* coordinate system established by that information. It is sometimes difficult to determine this direction, especially in the initial stages of early tsunami warnings. To overcome this, we rotate the equation from the *x–y–z* coordinate system to the *n–e–z* coordinate system and assume that the tsunami propagation direction is θ (figure 1*b*), then the horizontal components of equation (2.2) become


(2.3) $$\begin{array}{r} \begin{array}{r} \left\{ \begin{array}{ll} \left(\frac{\partial^{2}}{\partial y^{2}}+\frac{\partial^{2}}{\partial z^{2}}\right) & b_{y}\;\sin\theta =\sigma_{oc}\mu_{0}\frac{\partial b_{y}}{\partial t}\;\sin\theta +\sigma_{oc}\mu_{0}\frac{\partial }{\partial z}(-v_{y}\;\sin\theta F_{z}+v_{z}F_{y}\;\sin\theta )\\ \left(\frac{\partial^{2}}{\partial y^{2}}+\frac{\partial^{2}}{\partial z^{2}}\right) & b_{y}\;\cos\theta =\sigma_{oc}\mu_{0}\frac{\partial b_{y}}{\partial t}\;\cos\theta +\sigma_{oc}\mu_{0}\frac{\partial }{\partial z}(-v_{y}\;\cos\theta F_{z}+v_{z}F_{y}\;\cos\theta ). \end{array}\right. \end{array} \end{array}$$

Using $b_{y}\;\sin\theta =b_{n}$, $b_{y}\;\cos\theta =b_{e}$, $v_{y}\;\sin\theta =v_{n}$ and $v_{y}\;\cos\theta =v_{e}$ gives


(2.4) $$\begin{array}{r} \begin{array}{r} \left\{ \begin{array}{ll} \left(\frac{\partial^{2}}{\partial n^{2}}+\frac{\partial^{2}}{\partial e^{2}}+\frac{\partial^{2}}{\partial z^{2}}\right) & b_{n}=\sigma_{oc}\mu_{0}\frac{\partial b_{n}}{\partial t}+\sigma_{oc}\mu_{0}\frac{\partial }{\partial z}(-v_{n}F_{z}+v_{z}F_{y}\;\sin\theta )\\ \left(\frac{\partial^{2}}{\partial n^{2}}+\frac{\partial^{2}}{\partial e^{2}}+\frac{\partial^{2}}{\partial z^{2}}\right) & b_{e}=\sigma_{oc}\mu_{0}\frac{\partial b_{e}}{\partial t}+\sigma_{oc}\mu_{0}\frac{\partial }{\partial z}(-v_{e}F_{z}+v_{z}F_{y}\;\cos\theta ), \end{array}\right. \end{array} \end{array}$$

where the Laplacian operator becomes $\frac{\partial^{2}}{\partial n^{2}}+\frac{\partial^{2}}{\partial e^{2}}+\frac{\partial^{2}}{\partial z^{2}}$.

It is important to note that in the multiplication of sinθ and cosθ with $b_{y}$ in the partial derivative with respect to time, ∂/∂t, the tsunami propagation direction is assumed to remain constant over time. However, tsunami waves often consist of multiple directional components owing to reflections from islands or continents. Additionally, tsunamis generated by earthquakes with multiple faults, which is common, may include components propagating in different directions. Therefore, equation (2.4) essentially represents only a single component of a tsunami. The complete equation should account for the cumulative addition of components propagating in different directions, expressed as $b=\sum b_{i}(\theta_{i})$. Deriving an analytical solution for this scenario is challenging. In this study, we address the issue by neglecting the $v_{z}F_{y}$ term in equation (2.4), as discussed in §2*b*. At this stage, we focus on the case of a tsunami with a single directional component. We also assume that θ is independent of z to multiply sinθ or cosθ within the parentheses on the right-hand side of the equation. This assumption implies that the wave motion direction remains consistent vertically. As shallow water waves, tsunamis generally satisfy this condition.

A linear dispersive wave equation is used to represent tsunami particle motion [14]:


(2.5) $$\begin{array}{r} \left\{ \begin{array}{l} v_{y}=\eta \omega \frac{\cosh[k(z-H)]}{\sinh(kH)}e^{i(k\cdot y-\omega t)}\\ v_{z}=-i\eta \omega \frac{\sinh[k(z-H)]}{\sinh(kH)}e^{i(k\cdot y-\omega t)}, \end{array}\right. \end{array}$$

which is a wave equation in the *x–y–z* coordinate system. To use this in equation (2.3), we rotate it into the *n–e–z* coordinate system [14, exercise 7.2]:


(2.6) $$\begin{array}{r} \left\{ \begin{array}{l} v_{n}=\eta \omega \;\sin\theta \frac{\cosh[k(z-H)]}{\sinh(kH)}e^{i(k\cdot y-\omega t)}\\ v_{e}=\eta \omega \;\cos\theta \frac{\cosh[k(z-H)]}{\sinh(kH)}e^{i(k\cdot y-\omega t)}\\ v_{z}=-i\eta \omega \frac{\sinh[k(z-H)]}{\sinh(kH)}e^{i(k\cdot y-\omega t)}, \end{array}\right. \end{array}$$

where η, k=|k⋅y| and H are wave height, wave number in the tsunami direction and water depth, respectively. Equation (2.6) describes a linear dispersive wave with the y-direction rotated at an angle θ to the horizontal plane. Hence, the equations can represent a tsunami with a specific direction in the *n*–*e*–*z* coordinate system. The wave number k is in the tsunami y-direction, which is a vector combination of the wave numbers in the northward and eastward directions, denoted as $k=(k_{n},k_{e})$. However, these latter two individual components are difficult to determine without knowing the tsunami propagation direction. Therefore, the wave number k along the y-direction is preserved in the rotated equations because it can be determined using the dispersion relation


(2.7) $$\begin{array}{r} \begin{array}{r} \frac{w^{2}}{k^{2}}=\frac{g}{k}\tanh kH \end{array}. \end{array}$$

We substitute the velocities $v_{n}$, $v_{e}$ and $v_{z}$ in equation (2.4) by those used in the linear dispersive wave (equation (2.6)), and assume that the induced magnetic fields $b_{n}$ and $b_{e}$ share the same w-harmonic term, $e^{i(k\cdot y-\omega t)}$, as the velocity field. Under this assumption, we have $\frac{\partial b}{\partial t}=-i\omega b$ and $(\frac{\partial^{2}}{\partial n^{2}}+\frac{\partial^{2}}{\partial e^{2}})b=(-{k_{n}}^{2}-{k_{e}}^{2})b=-k^{2}b,$ where the wavenumber k is straightforward to obtain. Accordingly, we have


(2.8) $$\begin{array}{ll} & \begin{array}{r} \left\{ \begin{array}{ll} \left(-k^{2}+\frac{\partial^{2}}{\partial z^{2}}\right) & b_{n}=-i\omega \sigma_{oc}\mu_{0}b_{n}+\sigma_{oc}\mu_{0}\frac{\partial }{\partial z}(-\eta \omega \;\sin\theta \frac{\cosh[k(z-H)]}{\sinh(kH)}F_{z}-i\eta \omega \frac{\sinh[k(z-H)]}{\sinh(kH)}F_{y}\;\sin\theta )\\ \left(-k^{2}+\frac{\partial^{2}}{\partial z^{2}}\right) & b_{e}=-i\omega \sigma_{oc}\mu_{0}b_{e}+\sigma_{oc}\mu_{0}\frac{\partial }{\partial z}(-\eta \omega \;\cos\theta \frac{\cosh[k(z-H)]}{\sinh(kH)}F_{z}-i\eta \omega \frac{\sinh[k(z-H)]}{\sinh(kH)}F_{y}\;\cos\theta ) \end{array}\right. \end{array}\\ & \;⟹\;\\ & \begin{array}{r} \left\{ \begin{array}{ll} \left[\frac{\partial^{2}}{\partial z^{2}}-(k^{2}-i\omega \sigma_{oc}\mu_{0})\right] & b_{n}=\sigma_{oc}\mu_{0}\frac{\partial }{\partial z}(-\eta \omega \;\sin\theta \frac{\cosh[k(z-H)]}{\sinh(kH)}F_{z}-i\eta \omega \frac{\sinh[k(z-H)]}{\sinh(kH)}F_{y}\;\sin\theta )\\ \left[\frac{\partial^{2}}{\partial z^{2}}-(k^{2}-i\omega \sigma_{oc}\mu_{0})\right] & b_{e}=\sigma_{oc}\mu_{0}\frac{\partial }{\partial z}(-\eta \omega \;\cos\theta \frac{\cosh[k(z-H)]}{\sinh(kH)}F_{z}-i\eta \omega \frac{\sinh[k(z-H)]}{\sinh(kH)}F_{y}\;\cos\theta ). \end{array}\right. \end{array} \end{array}$$

The amplitudes of wave velocity on the surface (z=0) are


$$\begin{array}{r} \begin{array}{r} \left\{ \begin{array}{l} v_{n\_\text{Sur}}=\eta \omega \;\sin\theta \frac{\cosh(kH)}{\sinh(kH)}\\ v_{e\_\text{Sur}}=\eta \omega \;\cos\theta \frac{\cosh(kH)}{\sinh(kH)}. \end{array}\right. \end{array} \end{array}$$

Dividing the upper and lower expressions of equation (2.8) by $v_{n\_\text{Sur}}$ and $v_{e\_\text{Sur}}$, respectively, we obtain


(2.9) $$\begin{array}{r} \begin{array}{r} \left\{ \begin{array}{ll} \left[\frac{\partial^{2}}{\partial z^{2}}-(k^{2}-i\omega \sigma_{oc}\mu_{0})\right] & \frac{b_{n}}{v_{n\_\text{Sur}}}=\sigma_{oc}\mu_{0}\frac{\partial }{\partial z}(-\frac{\cosh[k(z-H)]}{\cosh(kH)}F_{z}-i\frac{\sinh[k(z-H)]}{\cosh(kH)}F_{y})\\ \left[\frac{\partial^{2}}{\partial z^{2}}-(k^{2}-i\omega \sigma_{oc}\mu_{0})\right] & \frac{b_{e}}{v_{e\_\text{Sur}}}=\sigma_{oc}\mu_{0}\frac{\partial }{\partial z}(-\frac{\cosh[k(z-H)]}{\cosh(kH)}F_{z}-i\frac{\sinh[k(z-H)]}{\cosh(kH)}F_{y}). \end{array}\right. \end{array} \end{array}$$

It can be seen that the two expressions in equation (2.9) are actually the same equation. As a result, the ratios between the induced magnetic field and surface velocity are equal:


(2.10) $$\begin{array}{ll} & \begin{array}{r} \frac{b_{n}}{v_{n\_\text{Sur}}}=\frac{b_{e}}{v_{e\_\text{Sur}}} \end{array}\\ & \Rightarrow \\ & \begin{array}{r} \frac{b_{n}}{b_{e}}=\frac{v_{n\_\text{Sur}}}{v_{e\_\text{Sur}}}=\tan\theta \end{array}. \end{array}$$

This suggests that the direction of the horizontal tsunami magnetic field is the same as the tsunami propagation direction. As a result, the tsunami magnetic field can be used to measure the tsunami propagation direction as indicated by Toh *et al*. [6].

### (b) Equations for estimating tsunami horizontal velocities

Equation (2.9) provides a relation between the tsunami magnetic field and its horizontal velocity field. It can be written as


(2.11) $$\begin{array}{r} \begin{array}{r} (\frac{\partial^{2}}{\partial z^{2}}-\alpha_{oc}^{2})\frac{b_{*}}{v_{*\_\text{Sur}}}=\frac{\sigma_{oc}\mu_{0}}{\cosh(kH)}\frac{\partial }{\partial z}\{-\cosh[k(z-H)]F_{z}-i\sinh[k(z-H)]F_{y}\}, \end{array} \end{array}$$

where $\frac{b_{*}}{v_{*\_\text{Sur}}}$ represent either $\frac{b_{n}}{v_{n\_\text{Sur}}}$ or $\frac{b_{e}}{v_{e\_\text{Sur}}}$, which are equal in value; and $\alpha_{oc}=\sqrt{k^{2}-i\omega \mu_{0}\sigma_{oc}}$. If the tsunami propagation direction is given, we can calculate $F_{y}$ which is the magnitude of the Earth’s magnetic field pointing in that direction and solve equation (2.11). The result has the form


(2.12) $$\begin{array}{r} \begin{array}{r} \frac{b_{*}(z)}{v_{*\_\text{Sur}}}=A(z,\omega ,\theta )e^{i\phi (z,\omega ,\theta )} \end{array}, \end{array}$$

which is a transfer function between the horizontal components of the tsunami magnetic field at a water depth z and the tsunami surface velocity, where A is amplitude response and ϕ is phase response. In other words, if horizontal tsunami magnetic field data are observed at water depth z (e.g. on the seafloor z=H), the tsunami surface horizontal velocity field can be calculated (or vice versa). Also, the linear dispersive wave equation (equation (2.6)) allows us to estimate the whole horizontal velocity field under the surface using surface velocity.

Equation (2.11) is suitable for tsunamis with a single-directional component. For tsunamis with multiple-directional components, a rigorous approach involves decomposing the horizontal magnetic field into different components based on the various directions of motion. Each component is then converted into the corresponding horizontal tsunami velocity using specific transfer functions for each direction, and these velocity components are summed to form the complete horizontal tsunami velocity field. However, this method is not recommended owing to its complexity and the challenges it presents.

A simpler approach is to obtain a representative tsunami propagation direction by analysing the direction of the horizontal magnetic field over a period of time; equation (2.10) shows that the horizontal magnetic field aligns with the tsunami’s motion direction. However, for early tsunami forecasting, it is crucial to obtain the velocity field as quickly as possible, and analysing the motion direction requires time. Therefore, we propose an alternative method, which ignores the $\sinh[k(z-H)]F_{y}$ term inside the braces on the right-hand side of equation (2.11). This allows us to derive a transfer function that does not depend on the tsunami motion direction.

We note that different tsunami directions have distinct transfer functions because, in equation (2.11), they have different $F_{y}$ components, which correspond to the geomagnetic field components aligned with the tsunami propagation direction. By comparing the amplitudes of the two terms on the right-hand side of the equation, which is the ratio between $v_{y}F_{z}$ and $v_{z}F_{y}$ ($v_{y}F_{z}\text{:}v_{z}F_{y}=\cosh[k(z-H)]F_{z}\text{:}\sinh[k(z-H)]F_{y}$), we find that for a tsunami, which behaves as a shallow wave, $v_{y}\gg v_{z}$. Thus, the $F_{y}$ term can be disregarded. However, at the magnetic equator, where $v_{y}F_{z}$ approaches zero, the $v_{z}F_{y}$ term becomes dominant, potentially introducing significant errors. The uncertainty caused by neglecting the $v_{z}F_{y}$ term is discussed in §5*b*. Nonetheless, the primary conclusion is that this term is generally negligible in most cases. This leads to an equation where the tsunami propagation direction is not required:


(2.13) $$\begin{array}{r} \begin{array}{r} (\frac{\partial^{2}}{\partial z^{2}}-\alpha_{oc}^{2})\frac{b_{*}^{\prime }}{v_{*\_\text{Sur}}^{\prime }}=\frac{\sigma_{oc}\mu_{0}}{\cosh(kH)}\frac{\partial }{\partial z}\{-\cosh[k(z-H)]F_{z}\}. \end{array} \end{array}$$

Its result has this form


(2.14) $$\begin{array}{r} \begin{array}{r} \frac{b_{*}^{\prime }(z)}{v_{*\_\text{Sur}}^{\prime }}=A(z,\omega )e^{i\phi (z,\omega )}, \end{array} \end{array}$$

which is also a transfer function similar to equation (2.12), but it is unaffected by the direction of tsunami propagation θ. This transfer function yields the same result for tsunamis of different directions, so it is not necessary to distinguish between different tsunami directions or to analyse the tsunami direction. Instead, we can directly convert the tsunami horizontal magnetic field into the horizontal tsunami velocity field.

**Figure 2.**
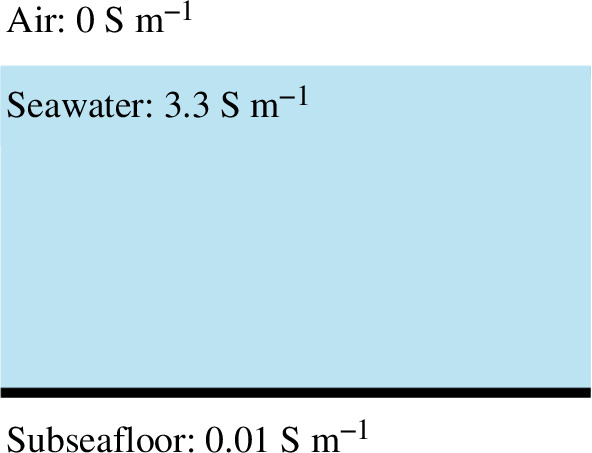
The three-layer model for solving equations (2.11) and (2.13)

Equations (2.11) and (2.13) were solved in a horizontal three-layer model (figure 2). The upper layer is an insulator half-space air layer. A global mean ocean conductivity of 3.3 S m^−1^ was used for the seawater layer [15]. Based on the work of Shimizu & Utada [16] and our test results, the subseafloor conductivity structure has minimal effect on the tsunami magnetic field. Therefore, we modelled it as a homogeneous half-space layer with a conductivity of 0.01 S m^−1^, consistent with previous studies (e.g. [17]).

The analytical solution to equation (2.11) in the three-layer model is


(2.15) $$\begin{array}{r} \begin{array}{rl} \frac{b_{*}(z)}{v_{*\_\text{Sur}}}= & A(z,\omega ,\theta )e^{i\phi (z,\omega ,\theta )}\\ = & C_{1}(\omega ,\theta )\frac{\alpha_{oc}}{k}e^{\alpha_{oc}z}-C_{2}(\omega ,\theta )\frac{\alpha_{oc}}{k}e^{-\alpha_{oc}z}\\ & +\frac{ikP}{\cosh(kH)}\cdot \left\{i\sinh[k(z-H)]F_{z}-\cosh[k(z-H)]F_{y}(\theta )\right\}, \end{array} \end{array}$$

where $P=\frac{\sigma_{oc}\mu_{0}}{k^{2}-\alpha_{oc}^{2}}$. The terms $C_{1}(\omega ,\theta )$ and $C_{2}(\omega ,\theta )$ are given by the following equations:


$$\begin{array}{r} \begin{array}{r} \begin{array}{rl} C_{1} & (\omega ,\theta )=\\ & \frac{\left(k+\alpha_{oc})\cdot [\frac{Pk}{\cosh(kH)}\cdot (kF_{y}-\alpha_{\text{sed}}F_{z})]-(\alpha_{\text{sed}}-\alpha_{oc})e^{-\alpha_{oc}H}\cdot \{-Pk^{2}\cdot (F_{y}+iF_{z})\cdot [1+\tanh(kH)]\right\}}{(k+\alpha_{oc})(\alpha_{\text{sed}}+\alpha_{oc})e^{\alpha_{oc}H}-(k-\alpha_{oc})(\alpha_{\text{sed}}-\alpha_{oc})e^{-\alpha_{oc}H}},\\ C_{2} & (\omega ,\theta )=\\ & \frac{\left(k-\alpha_{oc})\cdot [\frac{Pk}{\cosh(kH)}\cdot (kF_{y}-\alpha_{sed}F_{z})]-(\alpha_{\text{sed}}+\alpha_{oc})e^{\alpha_{oc}H}\cdot \{-Pk^{2}\cdot (F_{y}+iF_{z})\cdot [1+\tanh(kH)]\right\}}{(k-\alpha_{oc})(\alpha_{\text{sed}}-\alpha_{oc})e^{-\alpha_{oc}H}-(k+\alpha_{oc})(\alpha_{\text{sed}}+\alpha_{oc})e^{\alpha_{oc}H}}, \end{array} \end{array} \end{array}$$

where $\alpha_{\text{sed}}=\sqrt{k^{2}-i\omega \mu_{0}\sigma_{\text{sed}}}$ is the conductivity of the subseafloor. The solution to equation (2.13) is


(2.16) $$\begin{array}{r} \begin{array}{rl} \frac{b_{*}^{\prime }(z)}{v_{*\_\text{Sur}}^{\prime }}= & A(z,\omega )e^{i\phi (z,\omega )}\\ = & C_{1}^{\prime }(\omega )\frac{\alpha_{oc}}{k}e^{\alpha_{oc}z}-C_{2}^{\prime }(\omega )\frac{\alpha_{oc}}{k}e^{-\alpha_{oc}z}-\frac{kP}{\cosh(kH)}\cdot \sinh[k(z-H)]F_{z}, \end{array} \end{array}$$

where $C_{1}^{\prime }(\omega )$ and $C_{2}^{\prime }(\omega )$ are given by


$$\begin{array}{r} \begin{array}{r} \begin{array}{rl} C_{1}^{\prime } & (\omega )=\\ & \frac{\left(k+\alpha_{oc})\cdot [\frac{Pk}{\cosh(kH)}\cdot -\alpha_{\text{sed}}F_{z}]-(\alpha_{\text{sed}}-\alpha_{oc})e^{-\alpha_{oc}H}\cdot \{-Pk^{2}\cdot iF_{z}\cdot [1+\tanh(kH)]\right\}}{(k+\alpha_{oc})(\alpha_{\text{sed}}+\alpha_{oc})e^{\alpha_{oc}H}-(k-\alpha_{oc})(\alpha_{sed}-\alpha_{oc})e^{-\alpha_{oc}H}},\\ C_{2}^{\prime } & (\omega )=\\ & \frac{\left(k-\alpha_{oc})\cdot [\frac{Pk}{\cosh(kH)}\cdot -\alpha_{\text{sed}}F_{z}]-(\alpha_{\text{sed}}+\alpha_{oc})e^{\alpha_{oc}H}\cdot \{-Pk^{2}\cdot iF_{z}\cdot [1+\tanh(kH)]\right\}}{(k-\alpha_{oc})(\alpha_{\text{sed}}-\alpha_{oc})e^{-\alpha_{oc}H}-(k+\alpha_{oc})(\alpha_{\text{sed}}+\alpha_{oc})e^{\alpha_{oc}H}}. \end{array} \end{array} \end{array}$$

Equations (2.15) and (2.16) describe, respectively, the amplitude and phase relationship between the horizontal components of the tsunami magnetic field, $b_{n}$ and $b_{e}$, and the tsunami velocity field, $v_{n}$ and $v_{e}$, in the frequency domain. To calculate the tsunami’s horizontal velocities, $b_{n}$ and $b_{e}$ must first be transformed into the frequency domain. Then, using equations (2.15) and (2.16), the amplitude and phase of the tsunami’s horizontal velocities are calculated and finally transformed back into the time domain to obtain the tsunami’s horizontal velocity time series.

## 3. Data

**Figure 3.**
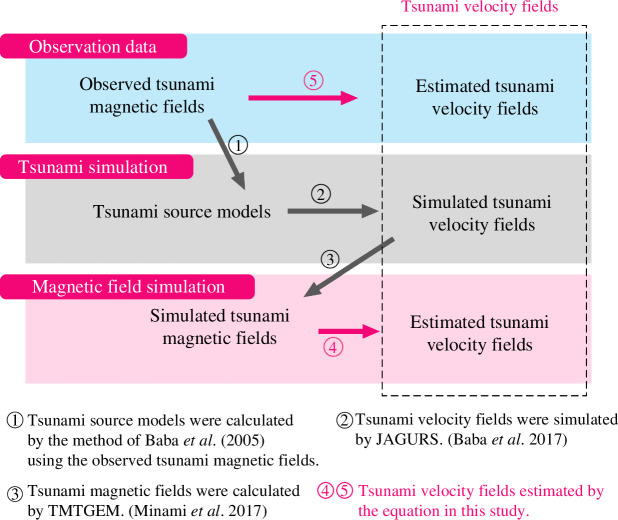
Three tsunami velocity field datasets: derived from an observed tsunami magnetic field (blue panel), a tsunami source model via tsunami simulation (grey panel), and a simulated tsunami magnetic field (red panel).

To examine our estimates of the tsunami horizontal velocity fields, we prepared three datasets: an observed tsunami magnetic field, a simulated tsunami magnetic field and a simulated tsunami velocity field (figure 3). The first two magnetic field datasets were converted into tsunami velocity fields using the method already described in this study. The last dataset was used to compare with the velocity fields derived from previous magnetic field data.

### (a) Observed tsunami magnetic field and tsunami pressure change data

**Figure 4.**
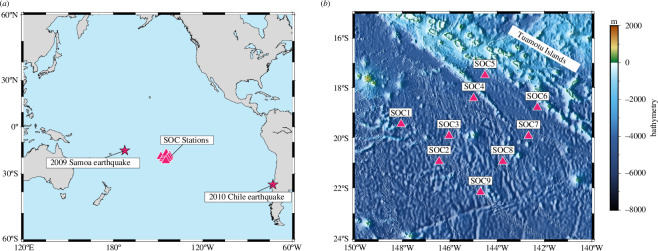
(*a*) The positions of the TIARES project OBEMs (red triangles) and the epicentres of the 2009 [11] Chile tsunamis (red stars). (*b*) Bathymetry of TIARES area. The figure was made using the Generic Mapping Tools version 6 (GMT6 [18]).

The observed tsunami magnetic field data were obtained from the seafloor ARray Experiment for the Society hotspot project (TIARES project [7]). Nine (SOC1–9) ocean-bottom electro-magnetometers (OBEMs) were deployed on the French Polynesian seafloor in the Pacific Ocean from 2009 to 2010 (figure 4). Additionally, a differential pressure gauge was installed on SOC8. These stations successfully recorded the magnetic and pressure change signals of the 2009 [11] Chile tsunamis.

The OBEMs recorded three components of the magnetic field, $b_{n}$, $b_{e}$ and $b_{z}$, using a 1 min sampling rate and a resolution of 0.01 nT [10]. To extract the magnetic signals of the 2009 [11] Chile tsunamis from the original observation data, the remote reference site method was used (e.g., [17]) to remove the external field and used bandpass filters with 3–30 and 3–45 min intervals, respectively (for more details, see [11]).

### (b) Simulated tsunami velocity fields

To reproduce tsunami velocity fields in the TIARES area, two tsunami source models were derived from tsunami magnetic field and pressure change data using Green’s function inversion method [19]. Details of these source models and comparisons between simulated magnetic fields and observations are provided in electronic supplementary material, figure S1. These models accurately explain the observed tsunami magnetic field (electronic supplementary material, figure S2) and are therefore believed to represent the tsunami velocity field in the TIARES area. However, since only TIARES data were used in the inversion, these models cannot account for observations outside the TIARES area or fully explain the characteristics of the tsunami source. In other words, the models are intended solely for estimating tsunami velocity fields within the TIARES area for this study.

The simulated tsunami velocity fields for the 2009 [11] Chile tsunamis were calculated using the JAGURS tsunami simulation code [20], which incorporates realistic bathymetry and tsunami dispersion.

### (c) Simulated tsunami magnetic fields

Using the two simulated tsunami velocity fields, the tsunami magnetic fields in the TIARES area for the 2009 [11] Chile tsunamis were simulated using the TMTGEM code [17]. This code incorporates realistic ocean bathymetry (SRTM15+ [21]), seawater conductivity [15] and the Earth’s magnetic field (IGRF−11 [22]).

## 4. Results

### (a) Comparison of the directions of horizontal tsunami magnetic field and tsunami propagation direction

**Figure 5.**
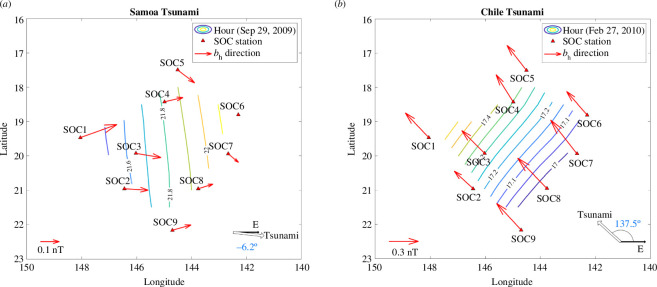
Horizontal tsunami magnetic field directions (red arrows), tsunami ETAs (coloured contour lines) and estimated tsunami propagation directions (numbers near hollow arrows in the bottom right) for the 2009 Samoa (*a*) and 2010 Chile (*b*) tsunamis. The numbers on the contour lines indicate the UTC times of the ETAs. The tsunami propagation direction is perpendicular to these lines.

To investigate the relationship between the directions of the tsunami propagation and the horizontal tsunami magnetic field, we derive two quantities from the observed tsunami magnetic fields. For the tsunami propagation direction, we first determine the arrival times of the tsunami magnetic field peaks at SOC1−9 (electronic supplementary material, figure S3) and then calculate the tsunami wavefront by fitting the following equation:


(4.1) $$\begin{array}{r} \begin{array}{r} t_{i}-t_{0}=\frac{\text{Distance}\;({\text{Lat}}_{i}-{\text{Lat}}_{0})}{U_{n}}+\frac{\text{Distance}\;({\text{Lon}}_{i}-{\text{Lon}}_{0})}{U_{e}} \end{array}, \end{array}$$

where $t_{0}$, ${\text{Lat}}_{0}$ and ${\text{Lon}}_{0}$ represent the tsunami arrival time, latitude and longitude, respectively, at the station where the tsunami first arrived (SOC1 for the 2009 Samoa tsunami and SOC9 for the 2010 Chile tsunami). The variables $t_{i}$, ${\text{Lat}}_{i}$ and ${\text{Lon}}_{i}$ indicate the arrival times and locations at the other SOC stations, respectively. The variables $U_{n}$ and $U_{e}$ denote the tsunami phase velocities in the northward and eastward directions, respectively ($U_{n}=\frac{\omega }{k\;\sin(\theta )}$ and $U_{e}=\frac{\omega }{k\;\cos(\theta )}$). Both $U_{n}$ and $U_{e}$ are greater than or equal to the tsunami phase velocity ($U=\frac{\omega }{k}$) and can become very large when the tsunami direction nearly aligns with the east (as in the 2009 Samoa tsunami) or is nearly perpendicular to it. Equation (4.1) was solved using QR decomposition; results are listed in table 1 and are illustrated in figure 5 as hollow arrows in the bottom right of each panel for the two tsunamis.

**Table 1.** Estimation results of tsunami propagation direction from tsunami arrival times.

event	$1/U_{e}$	$1/U_{n}$	$\arctan\left(U_{e}/U_{n}\right)$	RMSE^a^
2009 Samoa	0.001400 s m^−1^	−0.000152 s m^−1^	−6.2°	60.75 s
2010 Chile	−0.001000 s m^−1^	0.000916 s m^−1^	137.5°	39.98 s

^a^ RMSE= $\sqrt{\frac{1}{n}\sum_{i=1}^{n}{\left(t_{\text{estimated }}^{i}-t_{\text{observed }}^{i}\right)}^{2}}\;,$ where $t_{\text{estimated }}^{i}$ and $t_{\text{observed }}^{i}$ are the arrival time calculated by the fitting equation and read from the observation data at the SOCi station, respectively.

To compare the estimated tsunami directions with the horizontal tsunami magnetic components at each SOC station, coloured contours are plotted in figure 5 representing the tsunami estimated arrival times (ETAs), which were calculated by linear interpolation between the tsunami arrival times at all SOC stations. The tsunami propagation direction is perpendicular to these lines. The numbers on the lines indicate the time in decimal format, where, for example, 22.5 corresponds to 22:30 UTC. Using the time interval between the colour contour lines (6 min) as a reference, the root-mean-square errors (RMSEs) for the propagation direction estimations of the two tsunamis (table 1) are small, indicating that the estimated tsunami directions are reliable.

The horizontal magnetic component $b_{\text{h}}$ is a combination of the tsunami magnetic components $b_{n}$ and $b_{e}$. The $b_{\text{h}}$ values at each SOC station for the two tsunami events are shown as red arrows in figure 5, with the arrow lengths representing the magnitude of $b_{\text{h}}$. These magnitudes are approximately 0.1 nT for the 2009 Samoa tsunami and 0.3 nT for the 2010 Chile tsunami, as compared with the unit arrows in the bottom left of the panels. Note that the $b_{\text{h}}$ at the SOC6 station for the 2009 Samoa tsunami was excluded because the tsunami $b_{n}$ and $b_{e}$ components were unrecognizable in the observed data (electronic supplementary material, figure S3).

For the 2009 Samoa tsunami, the $b_{\text{h}}$ directions do not fully align with the tsunami propagation direction. Although they point eastward, they are not perpendicular to the ETA lines. In contrast, the tsunami $b_{\text{h}}$ directions for the 2010 Chile tsunami show a very accurate alignment with the tsunami propagation direction.

**Figure 6.**
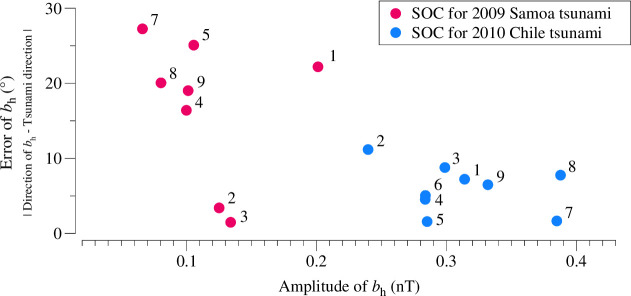
Errors in $b_{\text{h}}$ directions compared to the amplitude of $b_{\text{h}}$ for the 2009 Samoa (red circles) and 2010 Chile (blue circles) tsunamis. The error represents the absolute angular difference between the direction of tsunami $b_{\text{h}}$ at each SOC station and the tsunami propagation direction estimated from the ETAs at all stations.

One possible reason for the discrepancy in the Samoa event is the higher signal-to-noise ratio in the observed tsunami magnetic field data. We plot the angular differences between the tsunami $b_{\text{h}}$ direction and the estimated tsunami propagation direction against the amplitude of $b_{\text{h}}$ in figure 6. In the Samoa event, when $b_{\text{h}}$ amplitudes amplitudes are around 1 nT, the errors in $b_{\text{h}}$ direction are relatively large, approximately 20°–30°. In contrast, in the Chile event, where $b_{\text{h}}$ amplitudes are around 0.3 nT, the errors are much smaller, about 10°. This may indicate that a $b_{\text{h}}$ amplitude of 0.1 nT is insufficient to reliably indicate the tsunami propagation direction, whereas 0.3 nT can provide a more accurate estimation. This will be discussed in more detail in §5*a*.

### (b) Comparison of tsunami velocity fields estimation with and without tsunami propagation direction

In §2, we derived two equations for estimating tsunami horizontal velocity based on tsunami-induced magnetic fields. Equation (2.15) represents the analytical solution of Maxwell’s equations for unidirectional tsunamis while equation (2.16) provides a simplified approximation by neglecting the $v_{z}F_{y}(\theta )$ term for multidirectional tsunamis.

**Figure 7.**
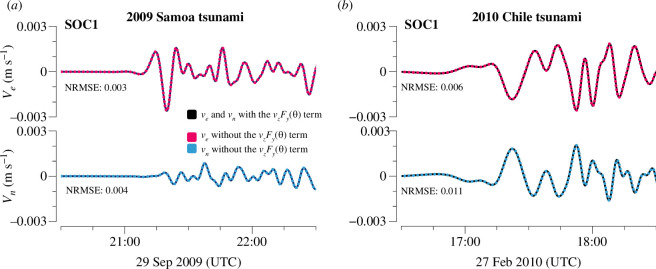
The tsunami horizontal velocities calculated using equations (2.15) and (2.16) from the simulated tsunami magnetic fields for the 2009 Samoa (*a*) and 2010 Chile (*b*) tsunamis.

To investigate the effect of ignoring the $v_{z}F_{y}(\theta )$ term, both equation (2.15) and (2.16) were used to estimate the horizontal tsunami velocities from the simulated tsunami magnetic fields of the 2009 [11] Chile tsunamis. Because the source areas of the 2009 Samoa tsunami and the 2010 Chile tsunami are very far apart, the tsunami velocity in the TIARES area can be observed to have a predominant direction. The tsunami propagation directions for equation (2.15) are −6.2${,}^{\circ }$ for the 2009 event and 137.5${,}^{\circ }$ for the 2010 event, which were estimated by fitting ETAs at all SOC stations using equation (4.1), as described in §4*a*. The Earth’s magnetic field intensities in the direction of tsunami propagation, $F_{y}(\theta )$, were determined to be 4027 and 20 511 nT for the 2009 [11] Chile tsunamis, respectively, according to the International Geomagnetic Reference Field-11th Generation (IGRF−11, [22]). The $F_{z}$ values for both events were −21 208 nT. The relatively small $F_{y}$ in the 2009 Samoa event is because the Earth’s magnetic field is directed approximately northward, so that when the tsunami propagated eastward, $F_{y}$ was relatively small. The calculated sea-surface tsunami velocities, $v_{n}$ and $v_{e}$, derived from tsunami $b_{n}$ and $b_{e}$ at the SOC1 station; the effects of both considering and ignoring the $v_{z}F_{y}(\theta )$ term are shown in figure 7. The normalized root-mean-square error (NRMSE) between the velocities with and without the $v_{z}F_{y}(\theta )$ term was also calculated.

Visually, the results from these two equations are nearly identical, and the NRMSE between them is very small. Additionally, the results at other SOC stations are similar to those at SOC1 shown in the figure. For the 2009 Samoa tsunami, which travelled eastward, the $F_{y}$ component was 4027 nT, and the $F_{z}$ component was −21 208 nT, giving a ratio of $|F_{y}/F_{z}|\approx 19$. This small ratio justifies the negligible effect of the tsunami magnetic field generated by $v_{z}F_{y}$. For the 2010 Chile event, $F_{y}$ and $F_{z}$ were 20 511 and −21 208 nT, respectively, indicating similar magnitudes. However, the results from equation (2.15) and equation (2.16) still show no significant difference. This suggests that even with a higher $F_{y}$, the effect of $v_{z}F_{y}$ remains minimal. The minimal differences between the results confirm that $v_{z}F_{y}$ has a negligible effect in both events, probably because the magnetic field generated by the tsunami’s vertical velocity is small compared with that generated by the horizontal velocity.

These results suggest that when $F_{y}$ is not significantly larger than $F_{z}$, a condition commonly observed in most regions on Earth, equation (2.16) provides similar accuracy to that of equation (2.15) for calculating tsunami horizontal velocities from the tsunami magnetic field. This indicates that the tsunami magnetic field generated by $v_{z}F_{y}$ can be considered negligible, making equation (2.16) very convenient for tsunami early warning, as it eliminates the need to estimate the tsunami propagation direction.

However, near the magnetic equator, where the vertical geomagnetic field is nearly zero, accounting for $F_{y}$ may become necessary. This will be further explored in §5*b*. For the TIARES data, equation (2.16) remains suitable, therefore, we will use it and disregard $v_{z}F_{y}$ to estimate tsunami velocities in the TIARES area here after.

### (c) Comparison of the estimated tsunami velocities from simulated tsunami magnetic fields with the tsunami velocity simulation results

When estimating tsunami horizontal velocities, several simplifying assumptions are made to solve Maxwell’s equations:

1. The seafloor is flat.
2. The seawater conductivity and the Earth’s magnetic field are uniform and constant.
3. The tsunami is a linear dispersive wave.

These simplifications may introduce inaccuracies that need further investigation. To address this, high-accuracy tsunami velocity simulations using JAGURS [20] and three-dimensional tsunami magnetic field simulations using TMTGEM [17] were used to evaluate the accuracy of tsunami velocity estimates, as described in §3*b*,*c*. This approach enabled us to assess the effect of the simplifications in equation (2.16) by comparing simulated tsunami velocities and magnetic fields with conditions that closely approximate real-world scenarios.

**Figure 8.**
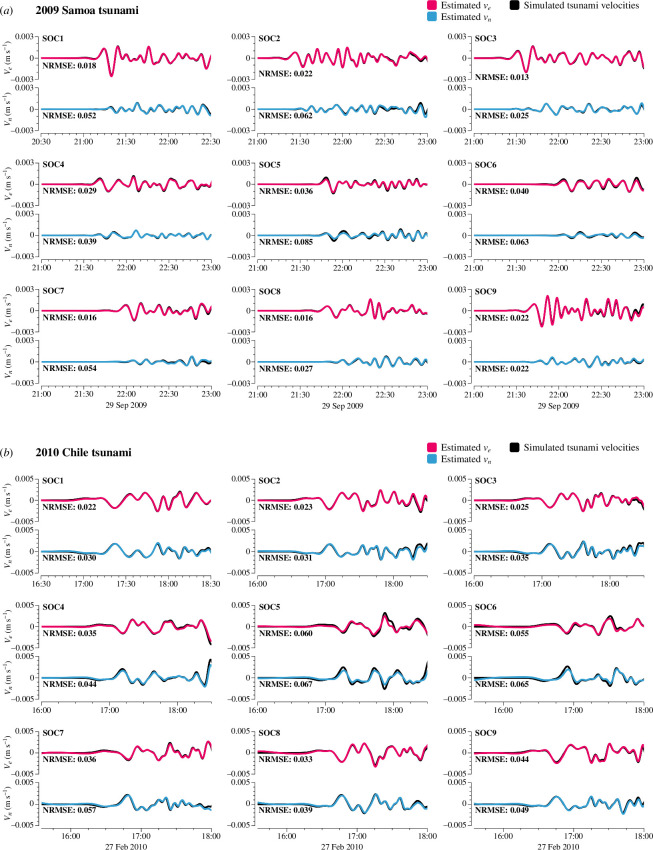
The tsunami horizontal velocities calculated by equation (2.16) from the simulated tsunami magnetic fields and simulated velocity fields from JAGURS for the 2009 Samoa (*a*) and 2010 Chile (*b*) tsunamis.

Using equation (2.16), we were able to estimate the tsunami horizontal velocity fields, $v_{n}$ and $v_{e}$, for the 2009 [11] Chile tsunamis at the SOC stations based on the simulated tsunami magnetic fields. These estimated velocity fields were then compared with those simulated by JAGURS, and the NRMSEs between them were calculated, as shown in figure 8.

This comparison corresponds to the processes indicated by arrows ③ and ④ in figure 3: the more realistic three-dimensional tsunami magnetic field simulation from TMTGEM versus the analytical solution using the simplified equation (2.16). The tsunami velocity fields calculated using equation (2.15) show excellent agreement with the three-dimensional simulation results, with favourable NRMSEs, confirming that our simplifications were reasonable. However, at SOC5 and SOC6, some discrepancies were observed, with slightly higher but still good NRMSEs. One hypothesis is that the complex bathymetry near the Tuamotu Islands (figure 4) may contribute to the complexity of tsunami motion at these stations, making the linear dispersive wave assumption less applicable. However, we do not have conclusive evidence to support this, and it remains an area for future study. In contrast, at stations on the mid-ocean seafloor, equation (2.16) provides accurate estimates of tsunami horizontal velocities based on the tsunami magnetic field.

### (d) Comparison of the estimated tsunami velocities from observed tsunami magnetic fields with the tsunami velocity simulation results

**Figure 9.**
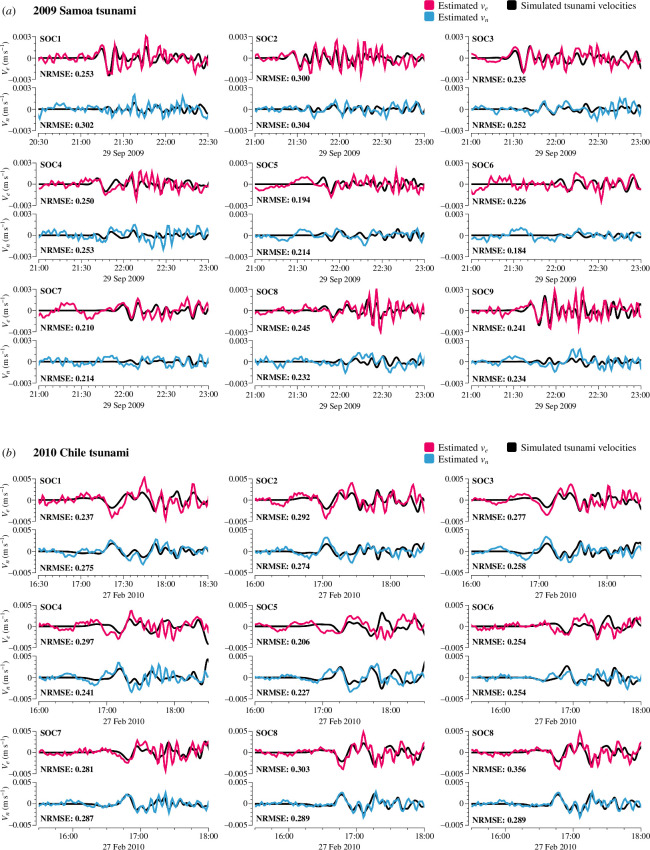
The tsunami horizontal velocities calculated using equation (2.16) from the observed tsunami magnetic fields and simulated velocity fields from JAGURS for the 2009 Samoa (*a*) and 2010 Chile (*b*) tsunamis.

Two sets of horizontal velocities for the 2009 [11] Chile events were also estimated from the tsunami data collected by the TIARES project. Using the observed tsunami $b_{n}$ and $b_{e}$ at the SOC stations, tsunami $v_{n}$ and $v_{e}$ were calculated using equation (2.16) (figure 9). This allows us to assess the applicability of the tsunami velocity estimation equation to actual data.

The simulated velocities used for this comparison are the same as those discussed in §4*b*, which involve three-dimensional simulations from JAGURS and TMTGEM. In figure 9, the estimated tsunami velocities derived from observational data are compared with these simulated velocities. This comparison assesses the accuracy of the simplified tsunami velocity estimation using equation (2.16) (represented by arrow ⑤ in figure 3), against the more complex, realistic three-dimensional simulation process that is designed to closely replicate real-world conditions (represented by arrows ①+② in figure 3).

As shown in figure 9, the observation data contain noise, unlike the simulated tsunami magnetic field data. This results in noticeably noisier velocities estimated from the observations and significantly higher NRMSEs compared with the simulation data. However, when focusing on the initial arrival peaks of the tsunami, where the simulated tsunami magnetic field closely aligns with the observed data (cf. electronic supplementary material, figure S2), there remains good agreement between the estimated and simulated tsunami velocities. This suggests that equation (2.15) is also applicable to observational data.

On the other hand, this highlights that noise in the observed tsunami magnetic data is a major source of error in practical velocity field estimation. While improving the signal-to-noise ratio in tsunami magnetic field observations is crucial for obtaining more accurate velocity fields, addressing this issue is beyond the scope of this paper.

## 5. Discussion

### (a) How large a tsunami magnetic field can provide an acceptable tsunami direction?

In §4*a*, we compared the tsunami propagation directions with the observed tsunami $b_{\text{h}}$ directions at SOC stations during the 2009 [11] Chile events. While the values of tsunami $b_{\text{h}}$ in the Chile tsunami clearly aligned with the tsunami propagation direction, the $b_{\text{h}}$ directions in the Samoa event did not. To explore whether this discrepancy could be attributed to noise in the tsunami magnetic field data, a Monte Carlo method with the following steps was used:

1. Generate a random tsunami propagation direction.
2. Calculate the amplitudes of tsunami $b_{n}$ and $b_{e}$ based on the tsunami direction and the amplitude of $b_{\text{h}}$.
3. Add random noise to $b_{n}$ and $b_{e}$, set at ±0.05 nT, based on the noise level observed in the magnetic signal before the tsunami’s arrival (refer to electronic supplementary material, figure S4).
4. Estimate the tsunami direction by calculating the direction of $b_{\text{h}}$ from the noise-added $b_{n}$ and $b_{e}$ values.
5. Determine the error as the difference between the estimated tsunami direction and the initial direction from step 1.

**Figure 10.**
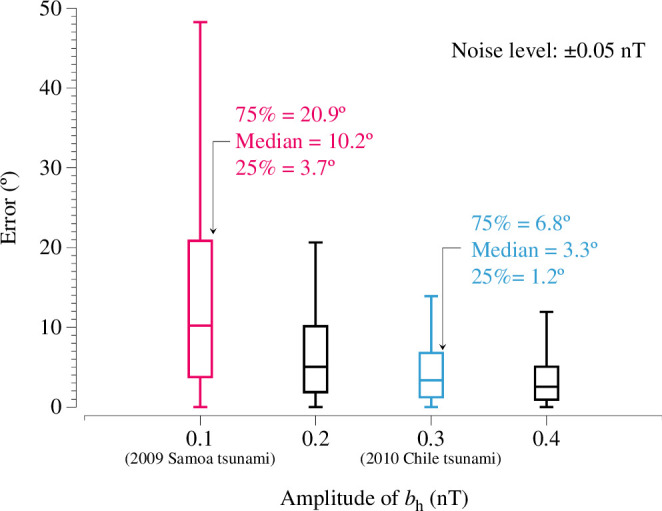
Box plot of the error distributions of tsunami propagation direction estimation with different amplitude of tsunami $b_{\text{h}}$ calculated by the Monte Carlo simulation. Each box represents 2000 samples, with the five horizontal lines in each box corresponding to the maximum value, 75th percentile, median, 25th percentile and minimum value, from top to bottom.

This process was repeated 2000 times for each of the four $b_{\text{h}}$ amplitudes: 0.1, 0.2, 0.3 and 0.4 nT. The error distributions for the different $b_{\text{h}}$ magnitudes are shown in figure 10. We see from figure 5 that the 2009 [11] Chile tsunamis can be represented by the 0.1 and 0.3 nT cases, respectively.

The height of the box in the figure represents the uncertainty in tsunami direction estimation. We use the 75th percentile of the error distribution as a measure of uncertainty, meaning that 75% of the estimation errors fall below this value. As shown in figure 10, the uncertainty for the 0.1 nT $b_{\text{h}}$ (representing the Samoa tsunami) is 20.9°, whereas for the 0.3 nT $b_{\text{h}}$ (representing the Chile tsunami) it is 6.8°. This explains the observations made relating to figure 6, where the error for the Samoa tsunami ranges from 20° to 30°, while for the Chile tsunami, it is predominantly less than 10°.

According to the Monte Carlo simulation, for a reliable estimation of tsunami propagation direction with an uncertainty of less than 10°, a tsunami $b_{\text{h}}$ of 0.2 nT or greater is required, assuming a noise level of ±0.05 nT.

### (b) The inaccuracy caused by ignoring the $v_{z}F_{y}(\theta )$ term

We derived two equations for estimating tsunami horizontal velocities from tsunami horizontal magnetic fields. Equation (2.15) includes the $v_{z}F_{y}(\theta )$ term, making it more accurate for unidirectional tsunamis. In contrast, while equation (2.16) omits this term, resulting in some accuracy loss, it can be used for multidirectional tsunamis. Simulation results for the SOC stations in the TIARES area show no apparent difference between these two equations (figure 7). However, as noted by Minami *et al*. [13], the $v_{z}F_{y}$ term can generate a significant magnetic signal when the horizontal geomagnetic field $F_{y}$ is larger than the vertical $F_{z}$ (e.g. near the geomagnetic equator).

To examine the differences between these two estimation equations in various geomagnetic field environments, such as different latitudes, and considering that the $v_{z}F_{y}(\theta )$ term varies with the tsunami propagation direction, another Monte Carlo simulation was designed:

1. Generate a random tsunami propagation direction.
2. Given a geomagnetic field, calculate the transfer functions for four tsunami periods: 10, 15, 20 and 25 min. Based on the wavelet spectrum analysis of Lin *et al*. [11], the major peak periods of the 2009 [11] Chile tsunami signals are 16 and 15 min, respectively. Therefore, these periods are selected.
3. Repeat the process 2000 times for each of the five latitudes: 60°N, 40°N, 20°N, 10°N and 0°N at 180°E (table 2). The geomagnetic fields at these positions were calculated using IGRF−13 [23] for a depth of 5000 m under water in 2020. These positions are categorized into three types: high-latitude ($F_{z}>2F_{\text{h}}$), mid-latitude ($F_{z}\approx F_{\text{h}}$) and low-latitude ($F_{\text{h}}>2F_{z}$) (electronic supplementary material, figure S5).

**Table 2.** Geomagnetic fields at simulated points for different latitudes.

	60°N 180°E	40°N 180°E	20°N 180°E	10°N 180°E	0°N 180°E
*F*_*e*_ (nT)	656.7	1995.6	4062.7	4968.5	5743.3
*F*_*n*_ (nT)	17 978.3	25 344.5	28 895.4	31 437.9	33 588.6
*F*_*z*_ (nT)	50 968.2	33 843.4	27 586.8	8382.7	−3063.6

Geomagnetic fields calculated using IGRF−13 in 2020 [23].

**Figure 11.**
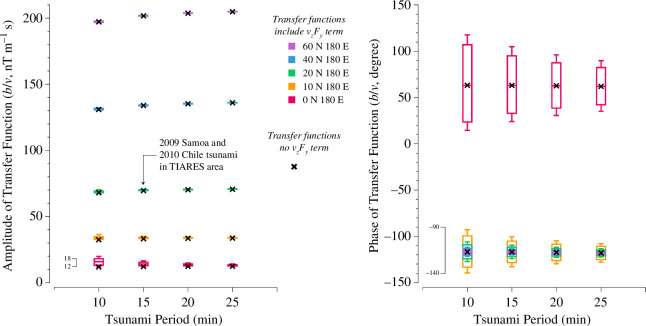
Box plots of the distributions of transfer functions including the $v_{z}F_{y}$ term (equation (2.15)) and transfer functions without the $v_{z}F_{y}$ term (equation (2.16)) at different latitudes. The distributions are based on Monte Carlo simulations. The left plot shows the amplitude response, while the right plot shows the phase response. Each box represents 2000 samples.

The distributions of amplitudes and phases of the transfer functions, including the $v_{z}F_{y}(\theta )$ term, are shown as box plots in figure 11. Because the value of $v_{z}F_{y}(\theta )$ varies with the tsunami propagation direction, a larger $v_{z}F_{y}(\theta )$ results in a broader distribution of transfer function values, as indicated by the larger box plots in figure 11. In contrast, transfer functions calculated without the $v_{z}F_{y}(\theta )$ term, based on the given geomagnetic fields and tsunami periods, do not fluctuate with changes in tsunami propagation direction and are represented as crosses in figure 11. Note that smaller amplitude responses in the transfer functions imply that the magnetic field generated by the tsunami motion is also smaller. In figure 11, it can be seen that the amplitude responses are very small in low-latitude regions, indicating that tsunamis produce weaker magnetic fields in these areas, making them more challenging to detect.

The amplitude response of the transfer function that includes the $v_{z}F_{y}$ term (equation (2.15)) shows a wider distribution range as the tsunami period decreases, indicating that the effect of $v_{z}F_{y}$ becomes stronger for shorter periods. In low-latitude areas, where the horizontal component of the Earth’s magnetic field ($F_{\text{h}}$) is greater than the vertical component ($F_{z}$), the distribution range of the transfer function is also broader. Conversely, when the $v_{z}F_{y}$ term is not considered, the amplitude responses are consistently equal to the minimum value observed when the term is included. This suggests that in such cases, the tsunami probably moved nearly vertically relative to $F_{\text{h}}$, minimizing the contribution of the $F_{y}$ component, which is aligned with the tsunami’s propagation direction.

Generally, the amplitude response distribution with $v_{z}F_{y}$ is very similar to the response without it. Even in extreme scenarios, such as a 10 min tsunami at the equator, the amplitude response variation of equation (2.15) is approximately 6 nT m${,}^{-1}$ s (75th percentile minus minimum value, as indicated by the bracket in figure 11). When multiplied by a tsunami velocity of 0.003 m s^−1^, the discrepancy between the amplitude responses of the two equations is approximately 0.018 nT, which is much smaller than the observational noise of 0.05 nT. This explains why the estimated tsunami velocities using equations (2.15) and (2.16) are almost identical in figure 7. In other words, the $v_{z}F_{y}(\theta )$ term does not significantly affect the tsunami magnetic amplitude response to tsunami velocity.

On the other hand, the $v_{z}F_{y}(\theta )$ term has a more significant effect on phase responses. When $F_{\text{h}}\gg F_{z}$ (e.g. at 0°N 180°E), $v_{z}F_{y}$ dominates the phase responses, even if $v_{z}$ is much smaller than $v_{y}$. In the right-hand panel of figure 11, the red boxes indicate phase response distribution ranges larger than 100° for 10 min tsunamis. In low-latitude areas (e.g. 10°N 180°E), the distribution range can still reach approximately 50°. When $F_{\text{h}}$ and $F_{z}$ are similar, the variation in phase response caused by $v_{z}F_{y}$ appears minimal.

In summary, ignoring the $v_{z}F_{y}$ term has the following effects on estimating tsunami horizontal velocities from magnetic fields: for amplitude estimation, there is only a trivial inaccuracy, while for phase estimation, there is a notable phase error in low-latitude regions. Additionally, the amplitudes of the transfer functions indicate that magnetic fields generated by tsunamis in these low-latitude regions are relatively weak. As a result, tsunami magnetic field detectors are probably not deployed in low-latitude areas owing to the challenge of detecting such weak signals. This practical constraint further justifies the omission of the $v_{z}F_{y}$ term in tsunami velocity estimation.

## 6. Conclusion

In this paper we describe the solution of the Maxwell’s equations coupled with linear dispersive wave equations, leading to the following findings:

1. The horizontal magnetic field direction aligns with the tsunami propagation direction.
2. The tsunami’s horizontal velocity can be calculated from its magnetic field.

For the first finding, we compared the directions of tsunami horizontal components $b_{\text{h}}$ and the tsunami propagation directions for the 2009 [11] Chile events using observation data from the TIARES project. In the case of the Chile tsunami, the directions of $b_{\text{h}}$ are consistent with the tsunami direction. However, for the Samoa tsunami, they are not. Monte Carlo analysis reveals that noise contributes to the inaccuracy observed in the Samoa event. The analysis also shows that the $b_{\text{h}}$ amplitude must be larger than 0.2 nT to accurately represent the tsunami propagation direction under a noise level of ±0.05 nT.

For the second finding, we developed two transfer functions: one that includes both $v_{h}F_{z}$ and $v_{z}F_{y}$ for unidirectional tsunamis, and another that excludes the smaller $v_{z}F_{y}$ term for for multidirectional tsunamis. Monte Carlo analysis indicates that these transfer functions are nearly identical in most cases. Significant inaccuracies in phase estimation occur only in low-latitude areas where $F_{y}$ is large if the $v_{z}F_{y}$ term is omitted.

Our results also demonstrate that the observation noise can lead to inaccuracies in tsunami direction and velocity estimates. Investigating these sources of error is important in understanding their effect on estimation results; however, this is beyond the scope of the current paper. To enhance the accuracy of the estimated velocity field, future efforts should focus on reducing noise in observed tsunami magnetic data.

Using tsunami magnetic data, we have demonstrated methods for obtaining tsunami velocity fields, which offer additional dimensions compared with sea-level change data. This approach allows for the inversion of more accurate tsunami source models. Furthermore, equation (2.16) enables the calculation of tsunami velocity fields without requiring the tsunami direction. As a result, OBEMs can be more effective than pressure gauges for obtaining tsunami propagation information, as they can determine both the tsunami motion direction and speed from a single monitoring station.

## Data Availability

The observed tsunami magnetic field data this study used are from the TIARES project (Suetsugu et al. 2012) and the author does not have permission to share. Requests to access the data should be directed to Dr. K. Baba at U. Tokyo through his homepage https://www.eri.u-tokyo.ac.jp/people/kbaba/. The tsunami velocity simulation code JAGURS in this study provided by Dr. T. Baba at Tokushima University is openly available from Github at [24]. The tsunami magnetic field simulation code TMTGEM in this study provided by Dr. T. Minami at Kobe University are openly available from Github at [25]. The tsunami source model for the 2009 Samoa and 2010 Chile tsunami and the control files for the tsunami velocity field and magnetic field simulations are available from Zenodo at [26]. Supplementary material is available online [27].
